# Weighted Risk Score-Based Multifactor Dimensionality Reduction to Detect Gene-Gene Interactions in Nasopharyngeal Carcinoma

**DOI:** 10.3390/ijms150610724

**Published:** 2014-06-13

**Authors:** Chao-Feng Li, Fu-Tian Luo, Yi-Xin Zeng, Wei-Hua Jia

**Affiliations:** 1 Department of Medical Statistics and Epidemiology, School of Public Health, Sun Yat-sen University, Guangzhou 510080, China; E-Mail: lichaofeng@sysucc.org.cn; 2 State Key Laboratory of Oncology in South China, Sun Yat-sen University Cancer Center, Guangzhou 510060, China; E-Mail: zengyx@sysucc.org.cn; 3 Department of Information Technology, Sun Yat-sen University Cancer Center, Guangzhou 510060, China

**Keywords:** gene-gene interaction, weighted risk score, multifactor dimensionality reduction, nasopharyngeal carcinoma

## Abstract

Determining the complex relationships between diseases, polymorphisms in human genes and environmental factors is challenging. Multifactor dimensionality reduction (MDR) has been proven to be capable of effectively detecting the statistical patterns of epistasis, although classification accuracy is required for this approach. The imbalanced dataset can cause seriously negative effects on classification accuracy. Moreover, MDR methods cannot quantitatively assess the disease risk of genotype combinations. Hence, we introduce a novel weighted risk score-based multifactor dimensionality reduction (WRSMDR) method that uses the Bayesian posterior probability of polymorphism combinations as a new quantitative measure of disease risk. First, we compared the WRSMDR to the MDR method in simulated datasets. Our results showed that the WRSMDR method had reasonable power to identify high-order gene-gene interactions, and it was more effective than MDR at detecting four-locus models. Moreover, WRSMDR reveals more information regarding the effect of genotype combination on the disease risk, and the result was easier to determine and apply than with MDR. Finally, we applied WRSMDR to a nasopharyngeal carcinoma (NPC) case-control study and identified a statistically significant high-order interaction among three polymorphisms: rs2860580, rs11865086 and rs2305806.

## 1. Introduction

Complex interactions among genes and environmental factors are known to play a role in common human disease etiology. However, the identification and characterization of gene-gene interactions for common complex human diseases remain a challenge for human geneticists. Traditional statistical methods are not well suited for detecting such interactions, especially when the data are highly dimensional (having many attributes or independent variables) or when interactions occur between more than two polymorphisms [1]. To address these issues, a variety of bioinformatics methods for identifying gene-gene interactions have been developed [2,3,4], and one such method is multifactor dimensionality reduction (MDR) [5,6,7,8,9,10,11,12]. The MDR method uses constructive induction to collapse high-dimensional genotype combinations into one-dimensional variables with two levels, high risk and low risk, using a threshold that is a function of the number of cases and controls [9]. Cross-validation and permutation testing are used to limit overfitting and false positives due to multiple testing. Since its initial description by Ritchie [5], many modifications and extensions to the MDR approach have been proposed. These include entropy-based interpretation methods [9], the use of odds ratios [13], log-linear methods [14], generalized linear models [15], methods for imbalanced data [16], permutation testing methods [17,18], methods for addressing missing data [19], parallel implementations [20,21], different evaluation metrics [22,23], methods for quantitative traits [24], balancing function methods [25] and the aggregated-multifactor dimensionality reduction method [26]. These extensions and modifications of MDR have each addressed different limitations, although classification accuracy is required for this approach. For example, when the proportion of cases in a study is extremely different from that of the real population, which is common in case-control studies, then the classification accuracy is difficult to determine and apply. Moreover, MDR methods cannot quantitatively assess the disease risk of genotype combinations; instead, they offer only a binary measure (high or low) of disease risk. Zhang and Liu proposed a Bayesian epistasis association mapping (BEAM) algorithm for identifying both single-marker and epistasis associations in population-based case-control studies [27]. The BEAM algorithm treats disease-associated markers and their interactions via a Bayesian partitioning model and computes, via Markov chain Monte Carlo, the posterior probability that each marker set is associated with the disease. In the simulated datasets, the BEAM method was considerably more powerful than existing methods for epistasis mapping. The research showed that the posterior probability could be an appropriate measure to assess epistatic interactions.

Nasopharyngeal carcinoma (NPC) is a squamous cell carcinoma that arises in the epithelial lining of the nasopharynx [28]. This neoplasm has a remarkable ethnic and geographic distribution, with a high prevalence in Southern China, Southeast Asia, Northern Africa and Alaska [29]. Hildesheima and Wang [30] reviewed genetic association studies of NPC and found consistent evidence for associations with a handful of genes, including immune-related HLA class I genes, the DNA repair gene, RAD51L1, cell cycle control genes MDM2 and TP53 and the cell adhesion/migration gene, MMP2. Two recent independent genome-wide association studies (GWAS) supported the HLA region as an NPC risk locus in Cantonese [31] and Taiwanese populations [32]. A Ras-independent pathway in the natural killer cell-mediated cytotoxicity (NK cell pathway) [33], regulates the role of NK cells in the immune response and involves HLA genes. Thus, we hypothesize that interactions among polymorphisms involved in the NK cell pathway may have a synergistic effect on the pathogenesis of NPC.

In this paper, we introduce a novel weighted risk score-based multifactor dimensionality reduction (WRSMDR) method for detecting and characterizing high-order gene-gene interactions in case-control studies. This WRSMDR method uses the Bayesian posterior probability of each genotype combination as a quantitative measure of disease risk and computes the proportion of each genotype combinations in all samples as the weight. WRSMDR exhaustively searches all possible combinations of polymorphisms to identify the one that can divide the samples into the best risk sub-groups. We first evaluated WRSMDR using simulated multi-locus data with epistatic effects and then compared it to the original MDR method. Next, we applied the WRSMDR method to identify multiple single-nucleotide polymorphisms (SNP) associated with nasopharyngeal carcinoma.

## 2. Results and Discussion

Section 3.1 describes the WRSMDR method in detail. The MDR software package was downloaded from [34]. To compare the WRSMDR and MDR methods, we defined four rates:

Specific detection rate = the proportion of simulated datasets in which the true model was detected as the overall best model [23].

Detection rate = the proportion of simulated datasets in which a multi-locus model, including the true model, was detected as the overall best model [23].

Error rate = the proportion of simulated datasets in which the overall best model did not include the true model.

No detection rate = the proportion of simulated datasets in which the method did not detect any statistically significant model.

### 2.1. Comparison of WRSMDR with MDR

We applied the WRSMDR and MDR methods to balanced and imbalanced simulated datasets, and the results are shown in Table 1 and Table 2, respectively. For the balanced datasets of two- and three-locus models, there was no significant difference between the detection rate and specific detection rate for the two methods (*p* > 0.05). The detection rates of the WRSMDR and MDR methods were 100% for the two- and three-locus simulated datasets. For the balanced four-locus datasets, the specific detection rates of the WRSMDR and MDR were 92% and 46%, respectively, and this difference was statistically significant (*p* < 0.05). The detection rates of the WRSMDR and MDR were 97% and 56%, respectively, and this difference was also statistically significant (*p* < 0.05). For the 100 balanced simulated four-locus datasets, the WRSMDR method did not produce statistically significant results in two datasets and produced error results in one dataset; the MDR method produced error results in 44 datasets. For the imbalanced datasets of three-locus models, there was no significant difference between the detection rate and the specific detection rate of the two methods (*p* > 0.05). For the imbalanced two- and four-locus models, the specific detection rate and the detection rate of the WRSMDR method were higher than the rates of the MDR method, and their respective differences were statistically significant (*p* < 0.05). In the simulated datasets, the WRSMDR method demonstrated reasonable power to identify high-order gene-gene interactions, and it was more effective than the MDR method at detecting four-locus models.

**Table 1:** Power comparison of the MDR and weighted risk score-based multifactor dimensionality reduction (WRSMDR) methods in balanced datasets.

Evaluation Indicator	Two-Locus		Three-Locus		Four-Locus	
	WRSMDR	MDR	WRSMDR	MDR	WRSMDR	MDR
Specific Detection Rate	0.87	0.83	0.74	0.83	0.92	0.46
Detection Rate	1	1	1	1	0.97	0.56
Error Rate	0	0	0	0	0.01	0.44
No Detection Rate	0	0	0	0	0.02	0

**Table 2:** Power comparison of the MDR and WRSMDR methods in imbalanced datasets.

Evaluation Indicator	Two-Locus		Three-Locus		Four-Locus	
	WRSMDR	MDR	WRSMDR	MDR	WRSMDR	MDR
Specific Detection Rate	0.96	0.61	0.57	0.66	0.94	0.68
Detection Rate	1	0.81	0.85	0.85	0.98	0.79
Error Rate	0	0.19	0.03	0.15	0.01	0.21
No Detection Rate	0	0	0.12	0	0.01	0

### 2.2. Application of WRSMDR to NPC Data

Table 3 summarizes the *p*-value, consistency and weighted risk score from the WRSMDR analysis of the NPC dataset for each two- to five-locus combinations. One three-locus model, which had the maximum consistency and the maximum weighted risk score, *p* < 0.001, was selected by using the WRSMDR method. This three-locus model included rs2860580, rs11865086 and rs2305806. Table 4 summarizes the disease probabilities estimated by Bayes’ posterior probability formula for each genotype combination of the three loci. The NPC risk of the different genotype combinations increased from approximately one-quarter of to three-times greater than the cumulative risk of the disease. The result showed that the influence on disease risk of a genotype at one locus is dependent upon the genotypes at the other two loci, which is evidence of gene-gene interactions.

We also applied the MDR method to explore the NPC data in a further step. The result is shown in Table 5. The best model detected by the MDR method was a three-locus combination, which was the same as the model detected by the WRSMDR method. For the four- and five-locus models, the results of the MDR method were different from the WRSMDR method. Because of the different evaluation measures for the best model, we cannot evaluate which method is better in real, unknown NPC data. However, the three-locus model, which was detected by two methods, may be meaningful for understanding the pathogenesis of nasopharyngeal carcinoma.

**Table 3:** Summary of the results for applying the WRSMDR method to the nasopharyngeal carcinoma (NPC) dataset.

Number of Locus	SNPs	Weighted Risk Score	Consistency	*p*
2	rs2860580-rs11865086	1.324	10	<0.001
3	rs2860580-rs11865086-rs2305806 *	1.332	10	<0.001
4	rs2860580-rs11865086-rs836475-rs4976028	1.266	4	<0.001
5	rs2860580-rs11865086-rs836475-rs4976028-rs6488297	1.236	7	<0.001

* The three-locus combination was selected as the best model by the WRSMDR method.

**Table 4:** Summary of the disease probability estimated using Bayes’ posterior probability.

Genotype Combination of the Three SNPs ^a^	Disease Probability ^b^	Fold Increase in Risk ^c^	Weight of Genotype ^d^
GG-CC-AG	0.00077	3.07	0.03
GG-CC-AA	0.00045	1.78	0.03
GG-AC-AA	0.00038	1.51	0.09
GG-AC-AG	0.00037	1.49	0.11
AG-CC-AG	0.00036	1.43	0.03
GG-AA-AA	0.00034	1.36	0.08
AG-AC-AA	0.00032	1.29	0.09
GG-AA-AG	0.00031	1.24	0.08
GG-AA-GG	0.00031	1.23	0.02
GG-AC-GG	0.00027	1.07	0.03
AG-CC-AA	0.00026	1.03	0.02
AG-AC-GG	0.00019	0.77	0.03
AG-AC-AG	0.00019	0.77	0.09
AG-AA-AG	0.00017	0.67	0.07
AG-AA-GG	0.00016	0.66	0.02
AG-AA-AA	0.00016	0.62	0.08
AA-AC-AG	0.00013	0.52	0.02
AA-AC-AA	0.00010	0.39	0.02
AA-AA-AG	0.00008	0.33	0.01
AA-AA-AA	0.00006	0.26	0.01

^a^ The three SNPs = rs2860580-rs11865086-rs2305806; ^b^ the disease probability is calculated by Bayes’ posterior probability formula, which represents the disease probability of an individual who carries a specific multi-locus genotype combination; ^c^ the fold increase in risk compared to the cumulative risk of NPC; ^d^ the weight of the genotype is the proportion of samples with the specific genotype combination.

The three SNPs are located in the HLA-A, MAPK3 and VAV1 genes, which play important roles in the NK cell pathway. NK cells are lymphocytes distinct from B- and T-cells that induce the perforin-mediated lysis of tumor cells and virus-infected cells, and the NK cell pathway regulates the role of NK cells in the immune response. The highest NPC risk among the three-locus genotype combinations was three times greater than the cumulative risk of the disease, which indicates that this pathway may be associated with NPC. To the best of our knowledge, this is the first report describing a three-locus interaction associated with NPC, and the results of this study therefore provide new insights into the pathogenesis of NPC.

**Table 5:** Summary of results applying the MDR method to the NPC dataset.

Number of Locus	SNPs	Prediction Error (%)	Cross-Validation Consistency	*p*
2	rs2860580-rs11865086	41.65	9/10	<0.001
3	rs2860580-rs11865086-rs2305806 *	40.48	10/10	<0.001
4	rs2860580-rs11865086-rs2305806-rs2115485	41.31	8/10	<0.001
5	rs2860580-rs11865086-rs2305806 -rs2115485-rs7166547	45.35	5/10	<0.022

* The three-locus combination was selected as the best model by MDR.

### 2.3. The Advantages and Limitations of WRSMDR

The WRSMDR method provides several advantages. First, similar to the original MDR method, the WRSMDR method is a non-parametric approach and assumes no particular genetic model; Second, the WRSMDR method provides a more robust quantitative measure of disease risk and reveals more information regarding the effect of certain genotype combinations on the disease risk, and this also represents an important difference from the MDR method, which only discretized the risk into high and low. Our results showed that the WRSMDR method had more power than the MDR method in detecting four-locus gene-gene interactions in the simulated datasets. For the balanced four-locus datasets, the specific detection rates of the WRSMDR and MDR method were 92% and 46%, respectively. For the imbalanced four-locus datasets, the specific detection rates of the WRSMDR and MDR method were 94% and 68%, respectively. The reason for this difference may lie in the fact that the MDR method is vulnerable to false positive and false negative errors when the sample size is small or when the number of simultaneously detected loci is large. In the case of this scenario, the number of cases and controls with a certain genotype combination is very small, and a small change in the frequency can change the classification to the opposite result. With the WRSMDR method, the quantitative measure of the disease risk was effected less than binary classification values in this scenario; Third, the WRSMDR method uses a weighted risk score rather than classification accuracy as the evaluation measure of the multi-locus interaction. The goal of MDR is to search a locus combination with maximum classification accuracy. For imbalanced datasets, classifiers seeking an accurate performance are not suitable. The imbalanced dataset can cause a seriously negative effect on classification accuracy [35]. For extreme situations, the maximum accuracy may occur when all of the data classify into the majority class. Foster gave a summary of the related issues in [36]. Weighted risk scores are based on the disease probability of genotype combinations. Disease probability was a continuous value, which avoids losing information that might be lost when probabilities are discretized into a binary classification value. Thus, the imbalanced dataset has less negative effect on the weighted risk score than classification accuracy. In the simulated imbalanced datasets, the detection rates of the WRSMDR method were higher than for the MDR method for the two- and four-locus model, and the WRSMDR method had a reasonable ability to deal with imbalanced data. Moreover, the WRSMDR method provides hypothesis testing for the best model by evaluating the weighted risk score, and only a locus combination with *p* < 0.01 may be selected. These measures provide WRSMDR with reasonable power to identify interactions among two or more loci in relatively small samples.

However, similar to MDR, WRSMDR has the limitation of being computationally intensive. A genome scan with hundreds or thousands of polymorphisms requires robust machine learning algorithms, as all of the possible multi-locus combinations cannot be exhaustively searched. This requirement, however, is a limitation of any multi-locus method that does not first condition on a particular locus showing an independent main effect (e.g., stepwise logistic regression) [5]. Consequently, we are currently evaluating machine learning strategies to optimize the selection of combinations of polymorphisms, such as greedy algorithms and parallel genetic algorithms.

## 3. Experimental Section

### 3.1. WRSMDR

With the WRSMDR method, the disease probability of an individual carrying a multi-locus genotype combination was used to assess the susceptibility of the genotype combination, which was denoted as *P*(*D*/*G*), with *D* and *G* representing the disease status and the genotype combination, respectively. *P*(*D*/*G*) is a measure of the disease risk for a genotype combination, with larger values corresponding to a higher risk of the genotype leading to disease. With a known cumulative risk of the disease and the genotype frequencies in cases and normal controls, this value can be calculated from the Bayesian posterior probability formula as follows:


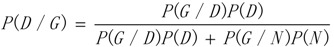
(1)

In this equation, *P*(*D*) represents the cumulative risk of the disease, *P*(*N*) = 1 − *P*(*D*). *P*(*G*/*D*) and *P*(*G*/*N*) represent the genotype combination frequencies in the case and control populations, respectively, and these values can be estimated from the case-control genotype dataset. The formula is as follows:


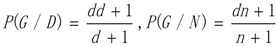
(2)

In this equation, *dd* and *dn* represent the number of individuals who carry the genotype combination in the case and control groups, respectively, and *d* and *n* represent the size of the case and control groups, respectively.

Suppose we want to investigate *k*-way gene-gene interactions. There are 3*^k^* different genotypic combinations at most. We denote *G_j_* (where *j* = 1, 2,...3*^k^*) as the genotype combinations of *k* loci, and we denote the relative risk of individuals with genotype *G_j_* as follows:


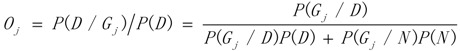
(3)

The extent of increased or decreased risk can be defined as follows:


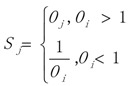
(4)

The weight of genotype *G_j_* can be defined as follows:


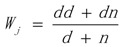
(5)

Based on the “common disease-common variant” hypothesis, we omit the genotype combinations with weights less than 0.01, and the weighted risk score is defined as follows:


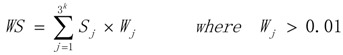
(6)

**Figure 1:**
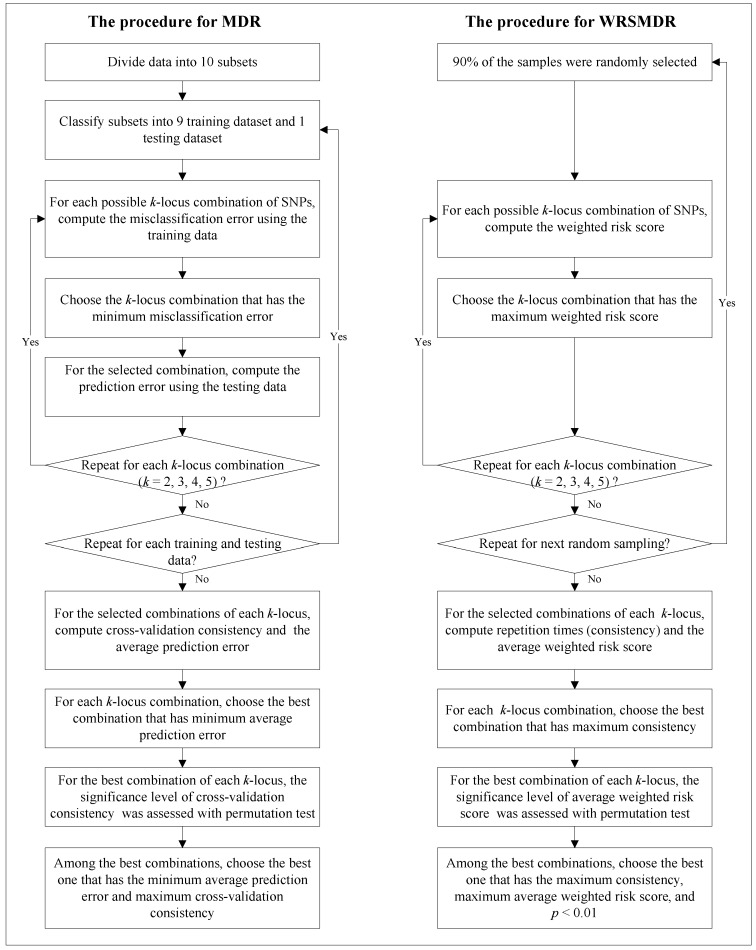
The procedure for the WRSMDR and MDR methods.

Instead of performing a direct search of the multi-locus combinations with the maximum score among the SNP set, which is liable to result in some false-positive loci in the detected multi-locus combinations, we used a random sampling method to repress noise in the identification of the susceptibility of multi-locus combinations. This procedure included three steps. In Step 1, 90% of the samples were randomly selected. The weighted risk scores of *k*-locus combinations (*k* ϵ {2,3,4,5}) were computed based on the randomly selected samples, and the combination with the maximum score within each of the *k*-locus combinations was selected. After ten repeats for each *k*-locus combination, the multi-locus combination that demonstrated the maximum consistency, that is, the one appearing most frequently, was chosen as the candidate susceptibility *k*-locus combination. The average score of each of the selected *k*-locus combinations was then computed; In Step 2, the significance level of each candidate *k*-locus combination was assessed with the permutation test. Specifically, for every 1000 permutation datasets simulating the null hypothesis of no association, the weighted risk score of the multi-locus combination was computed using the above method. The *p*-value was then determined according to the proportion of the permutation datasets with a greater weighted risk score than the average score. The null hypothesis was rejected when the Monte Carlo *p*-value derived from the permutation test was less than 0.01; In Step 3, among the candidate locus combinations, the one with *p* < 0.01, the maximum consistency, and the maximum average weighted risk score was selected as the final susceptibility multi-locus combination. Figure 1 describes the procedure of the WRSMDR and MDR methods. Additional details describing the MDR method are available in the literature [5,6,7,8,9,10].

### 3.2. Data Simulation

To evaluate the WRSMDR method, we simulated six sets of 100 replicates using three different multi-locus genetic models. Three sets were balanced, and the simulated dataset was composed of 400 cases and 400 controls. The other three sets are imbalanced, and the simulated dataset was composed of 1200 cases and 400 controls. All the genetic models and datasets were generated using the Genetic Architecture Model Emulator for Testing and Evaluating Software (GAMETES) [37]. In particular, GAMETES is designed to generate epistasis models that we refer to as pure and strict. Purely and strictly epistasis models constitute the most difficult type of disease association model to detect, as such associations may be observed only if all n-loci are included in the disease model. This requirement makes these types of models an attractive gold standard for simulation studies of complex multi-locus effects. The parameters of the three models are shown in Table 6. A large heritability implies a strong correlation between phenotype and genotype, such that loci with an effect on the trait can be more easily detected [38]. To demonstrate the detection ability of the WRSMDR method in difficult models, we selected a relatively small value of 0.05 for the heritability. Datasets simulated using GAMETES demonstrated two types of attributes (SNPs): predictive attributes and non-predictive attributes. Predictive attributes were those specified in the genetic model, whereas non-predictive attributes included all other attributes with no specified association with affected status (*i.e*., case or control). The six datasets consisted of 10 SNPs, including 2, 3 and 4 predictive SNPs and up to 8 randomly generated non-predictive SNPs.

**Table 6:** The parameter settings of the three models.

Parameters	Two-Locus Model	Three-Locus Model	Four-Locus Model
Number of predictive SNPs	2	3	4
Number of non-predictive SNPs	8	7	6
Heritability	0.05	0.05	0.05
MAF of predictive SNPs	0.2	0.2	0.2
MAF of non-predictive SNPs	(0.01~0.5)	(0.01~0.5)	(0.01~0.5)

MAF = minor allele frequency.

### 3.3. NPC Data

This NPC data were based on a large GWAS of NPC that was performed on Southern Chinese individuals by genotyping 620,901 SNPs in 1615 cases and 1025 controls of persons of Han Chinese descent from Guangdong and an additional 1008 Singapore Chinese controls, who share the same ancestral origin with Han Chinese individuals in Southern China [31]. After stringent quality control filtering, 464,328 autosomal SNPs in 1583 cases and 1894 controls (972 Guangdong subjects and 922 Singapore subjects) were retained for statistical analysis. Principal component analysis (PCA) showed that the 1583 cases and 972 Guangdong controls were genetically well matched, although the inclusion of the 922 Singapore controls caused moderate population stratification in the GWAS sample [31]. Consequently, only the genome-wide data for 1583 cases and 972 Guangdong controls of Guangdong subjects were included in this study. To extract the SNP data for the NK cell pathway, we performed three steps. In step 1, we identified the genes in the NK cell pathway based on the BioCarta database; In step 2, we selected the SNPs within 20-kb upstream or downstream of each gene’s coding region (National Center of Biotechnology Information’s human genome 18, hg18 database); In step 3, for each SNP selected in step 2, we performed genotype-phenotype association analysis using the Cochran-Armitage trend test with PLINK (v1.07) [39]. We selected the SNP with the maximum chi-square value to represent each gene. In this way, we obtained 19 SNPs involved in the NK cell pathway (see Table 7).

### 3.4. Data Analysis

Prior to applying WRSMDR to the NPC dataset, the method was evaluated using the simulated multi-locus datasets. For every 100 replicates generated by each of the three multi-locus epistasis models, we applied the WRSMDR algorithm as described in the subsection “WRSMDR”. An exhaustive search of all possible two- to five-locus models was performed. Then, the WRSMDR method was applied to the NPC dataset with the cumulative risk of the disease equal to 0.00025 [29]. Finally, we used a chi-square test to evaluate significant differences among the four rates between the WRSMDR and MDR methods.

**Table 7:** NK cell pathway SNPs involved in this study.

SNP	Chr.	Locus	MA	Chi-Square Value
rs2860580	6	HLA-A	A	89.95
rs11865086	16	MAPK3	C	14.96
rs4976028	5	PIK3R1	G	9.98
rs11150675	16	LAT	A	7.47
rs6488297	12	KLRC1	A	7.05
rs941831	10	ITGB1	G	5.88
rs836475	7	RAC1	A	4.80
rs2733840	12	KLRC4	G	3.02
rs2733840	12	KLRC3	G	3.02
rs10109834	8	PTK2B	C	2.71
rs2115485	9	SYK	A	2.68
rs2305806	19	VAV1	G	2.57
rs7166547	15	MAP2K1	A	2.35
rs744167	12	PTPN6	A	1.97
rs7301582	12	KLRC2	A	1.45
rs3019238	11	PAK1	G	1.23
rs7645550	3	PIK3CA	A	0.76
rs11214093	11	IL18	G	0.70
rs12310310	12	KLRD1	A	0.58
rs4780	15	B2M	G	0.23

Chr., chromosome; MA, minor allele.

## 4. Conclusions

In this study, we introduced WRSMDR as a method for detecting gene-gene interactions in case-control studies. We compared the WRSMDR and MDR methods in simulated datasets. Our result showed that the WRSMDR method had reasonable power to identify high-order interactions in simulated datasets. In particular, for the four-locus datasets, the detection rate and specific detection rate of the WRSMDR method were higher than the MDR method, whereas the error rate of the WRSMDR method was lower than the MDR method; these differences were statistically significant. The WRSMDR method was more effective than the MDR method at detecting four-locus models in the simulated datasets. Moreover, the WRSMDR method reveals more information regarding the effect of genotype combinations on the disease risk. We then applied WRSMDR to identify gene-gene interaction effects in the NK cell pathway related to the risk of NPC, and we found a statistically significant, high-order interaction among three polymorphisms. For ease of use, the source code and binaries are freely available for download at [40]. However, the WRSMDR method has the limitation of being computationally intensive, and we are currently exploring new strategies to optimize this approach.
